# Supplementary material for: Thermal Behavior and Pyrolysis Kinetics of Mushroom Residue with the Introduction of Waste Plastics
Source: Polymers (Basel). 2023 Sep 19;15(18):3824. doi: 10.3390/polym15183824 (PMC10534543; doi:10.3390/polym15183824)
Supplement: Supplementary file 1 [file polymers-15-03824-s001.zip › polymers-2573965-supplementary.pdf]

Supplementary materials

Table S1 Sample preparation

Sample name	Types of plastics	Ratio of MR and PE	Mass of MR (g)	Mass of plastics (g)
MR/PE-5:1		5:1	5	1
MR/PE-2:1		2:1	4	2
MR/PE-1:1	PE	1:1	3	3
MR/PE-1:2		1:2	2	4
MR/PE-1:5		1:5	1	5
MR/PP-1:1	PP	1:1	3	3
MR/PET-1:1	PET	1:1	3	3

Table S2 Samples for thermogravimetric analysis

	Heating rate (℃/min)	Name of sample
MR		MR-10 ℃/min
PE	10	PE-10 ℃/min
PP		PP-10 ℃/min
PET		PET-10 ℃/min
	10	MR/PE-1:1-10 ℃/min
	20	MR/PE-1:1-20 ℃/min
MR/PE-1:1	30	MR/PE-1:1-30 ℃/min
	40	MR/PE-1:1-40 ℃/min
MR/PE-5:1		MR/PE-5:1-10 ℃/min
MR/PE-2:1		MR/PE-2:1-10 ℃/min
MR/PE-1:2		MR/PE-1:2-10 ℃/min
MR/PE-1:5	10	MR/PE-1:5-10 ℃/min
MR/PP-1:1		MR/PEP-1:1-10 ℃/min
MR/PET-1:1		MR/PET-1:1-10 ℃/min

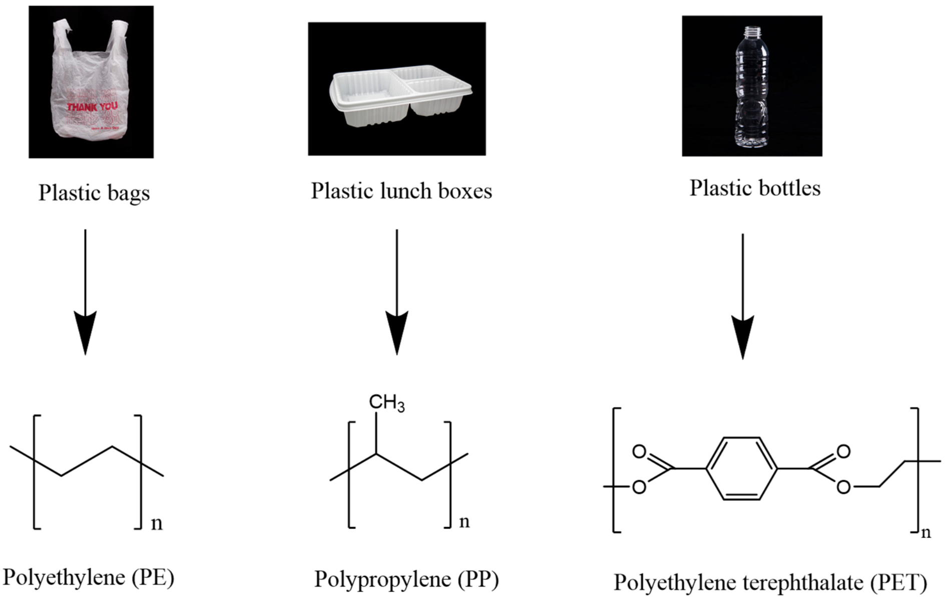

Figure S1. Chemical structure of PE, PP and PET.
